# Retraction Note: A biomimetic assay platform for the interrogation of antigen-dependent anti-tumor T-cell function

**DOI:** 10.1038/s42003-021-02569-1

**Published:** 2021-08-27

**Authors:** Jeremy To, Doug Quackenbush, Emily Rowell, Lilin Li, Connor Reed, Frederick Lo, Shane R. Horman

**Affiliations:** 1 Department of Functional Genomics, Genomics Institute of the Novartis Research Foundation (GNF), San Diego, CA USA; 2 Department of Online Screening, Genomics Institute of the Novartis Research Foundation (GNF), San Diego, CA USA; 3 Department of Cancer Therapeutics, Genomics Institute of the Novartis Research Foundation (GNF), San Diego, CA USA; 4 Department of Biotherapeutics and Biotechnology, Genomics Institute of the Novartis Research Foundation (GNF), San Diego, CA USA; 5 Department of Cheminformatics, Genomics Institute of the Novartis Research Foundation (GNF), San Diego, CA USA; 6 Present Address: Inhibrx, La Jolla, CA USA; 7 Present Address: Takeda Pharmaceuticals, San Diego, CA USA

**Keywords:** Tumour immunology, High-throughput screening, Cancer models, Tumour immunology

Retraction to: *Communications Biology* 10.1038/s42003-020-01565-1, published online 8 January 2021.

The authors are retracting this Article as inconsistencies were found between the work that was done and how the work and materials were described in the paper. These inconsistencies impact the accuracy of at least one figure and may put the conclusions of the Article into question. These data integrity issues undermine our full confidence in the integrity of the study and the authors therefore wish to retract the Article.

All the authors agree with the retraction.

